# From Mitochondria to Immunity: The Emerging Roles of Mitochondria‐Derived Vesicles and Small Extracellular Vesicles in Cellular Communication and Disease

**DOI:** 10.1002/jev2.70192

**Published:** 2025-11-11

**Authors:** Rostyslav Horbay, Vasyl Syrvatka, Artem Bedzay, Mikaela van der Merwe, Dylan Burger, Shawn T. Beug

**Affiliations:** ^1^ Apoptosis Research Centre Children's Hospital of Eastern Ontario Research Institute Ottawa Ontario Canada; ^2^ Centre for Infection, Immunity and Inflammation University of Ottawa Ottawa Ontario Canada; ^3^ Ottawa Institute of Systems Biology University of Ottawa Ottawa Ontario Canada; ^4^ Department of Biochemistry, Microbiology and Immunology University of Ottawa Ottawa Ontario Canada; ^5^ Genetic and Biotechnology Department Ivan Franko National University of Lviv Lviv Ukraine; ^6^ St. Luke's Hospital 1st Territorial Medical Association of the City of Lviv Lviv Ukraine; ^7^ Kidney Research Center Ottawa Hospital Research Institute University of Ottawa Ottawa Ontario Canada

**Keywords:** endosome, endosomal sorting complex required for transportation (ESCRT), lysosome, mitochondria‐derived vesicles (MDVs), mitophagy, multivesicular body (MVB), peroxisome, small extracellular vesicles (sEVs)

## Abstract

According to the endosymbiotic theory of mitochondrial origin, an α‐proteobacterium entered a prokaryotic cell and, through symbiosis, evolved into the mitochondria—the powerhouse of the cell. Like other bacteria, the α‐proteobacteria generate their own extracellular vesicles (EVs), a trait that was passed onto the mitochondria, enabling them to generate mitochondria‐derived vesicles (MDVs). MDVs, similar to small EVs (sEVs), are vesicles ranging from 30 to 200 nm in diameter and carry cargo for degradation by lysosomes and peroxisomes. MDVs share several features with sEVs, including targeted cargo degradation, biogenesis, packaging into multivesicular bodies, nucleic acid and protein transportation, induction of immune responses, and surface antigen presentation. MDVs may also be released from the cell in a manner similar to sEVs, potentially influencing intercellular communication and immune responses. Furthermore, the presence of MDVs presents opportunities for early disease detection, including neurodegenerative disorders and cancer. In this review, we explore the differences and similarities between MDVs and EVs, including their roles in immunity.

## 1 Introduction

Extracellular vesicle (EV) transport is an ancient and highly conserved mechanism of cellular communication (Mashburn and Whiteley 2005). Several types of EVs have been described, including apoptotic bodies (ApBs), microvesicles (MVs), ectosomes (ECs) and exosomes (EXs). These vesicles are distinguished by their modes of biogenesis, cargo composition, delivery mechanisms and uptake pathways (Lötvall et al. 2014). A more recently identified class of intracellular vesicles derived from mitochondria, termed mitochondria‐derived vesicles (MDVs), have been observed in a variety of eukaryotic cells ranging from plants to mammals (Soubannier, McLelland, et al. 2012; Sugiura et al. 2014; Yamashita et al. 2016). MDVs share several features with EVs, including specific cargo encapsulation, targeted delivery and vesicular structure.

MDVs have important roles in diverse cellular functions. Three major functions have been described for MDVs: protection of mitochondria from damaged proteins, presentation of mitochondrial antigens and regulation of cell homeostasis (Neuspiel et al. 2008; Matheoud et al. 2016). Consequently, disruption in MDV pathways is associated with several serious diseases, including autoimmune, neurodegenerative and cardiac disorders (Sugiura et al. 2014; Matheoud et al. 2016; McLelland et al. 2014). Understanding the mechanisms of MDVs and their role in immunity is thus critical for their potential diagnostic and therapeutic applications. In this review, we detail the mechanism of MDV biogenesis and compare MDVs to sEVs. We also discuss the evolutionary origins of these intracellular and extracellular vesicles, as well as their role in cargo transport. Further, we explore the shared and distinct roles of sEVs and MDVs in innate and adaptive immune responses in both physiological and pathological contexts, and provide future perspectives and challenges in this field.

## 2 sEV Biogenesis and the Role of ESCRT‐Dependent and ‐Independent Machinery

sEVs are a class of secreted vesicles described as 30–150 nm in size (Théry et al. 2018; Welsh et al. 2024). sEVs originate through two distinct mechanisms: (i) fusion of the multivesicular body (MVB) with the plasma membrane, which then release intraluminal vesicles (ILVs)—often termed EXs or (ii) via direct budding of vesicles from the plasma membrane, which are known as ectosomes (Théry et al. 2018; Horbay et al. 2022; Meldolesi 2018; Welsh et al. 2024). EXs harbour distinct surface markers and cargo due to their endosomal origin. Unlike MVs and ApBs, EXs transport specific biomolecules to recipient cells. Due to the overlap in size and the difficulty in isolating pure EVs, particularly when considering their biogenesis pathway, standardized guidelines recommend characterizing EVs based on specific protein markers (e.g., tetraspanins, miRNAs), size and morphology (Lötvall et al. 2014; Théry et al. 2018; Welsh et al. 2024). Because mitochondria were once independent organisms that can release EVs and that MDVs are capable of being secreted outside the cell, this vesicular class may fall within the scope to be classified as an EV.

sEV biogenesis is commonly described as Endosomal Sorting Complex Required for Transport (ESCRT)‐dependent or ‐independent, a distinction particularly relevant to EX formation. Besides sEV biogenesis, the ESCRT machinery has important roles in other cellular processes such as viral budding and cytokinesis. The ESCRT machinery consists of four core complexes (ESCRT‐0 to ‐III) and accessory proteins (e.g., RAB proteins, VPS4A, ALIX). The ESCRT system governs ILV formation and cargo sorting within MVBs, ultimately determining whether ILVs are secreted as EXs or degraded in lysosomes. In parallel, an ESCRT‐independent mechanism, such as those mediated by ceramide or RAB31, also contributes to EX formation. However, it must be kept in mind that EX biogenesis is best viewed as a continuum rather than a binary ESCRT‐dependent or ‐independent process (Horbay et al. 2022; Larios et al. 2020; Wei et al. 2021). We summarize the key differences between the ESCRT‐dependent and ‐independent pathways for EX generation in Table 1 (Horbay et al. 2022; Larios et al. 2020; Wei et al. 2021; Stoorvogel 2015; Trajkovic et al. 2008; Babst 2011) as several of these mechanisms and proteins are also implicated in MDV biogenesis.

**TABLE 1:** Comparative analysis of ESCRT‐dependent and ‐independent pathways.

Feature	ESCRT‐dependent pathway	ESCRT‐independent pathway
Initiator complex	ESCRT‐0 (Hrs/STAM1)	Ceramides, Rab31
Key proteins	TSG101, Alix, CHMPs, Vps proteins, Syntenin, Syndecans,	Rab31, nSMase2, Flotillin‐1/2
Membrane curvature and budding	CHMP4 spiral assembly, tetraspanins (CD63, CD81, CD82)	Ceramide‐induced curvature, lipid rafts
Cargo sorting	Ubiquitin‐dependent; Syntenin/Alix‐mediated	Ubiquitin‐independent; EGFR/Rab31‐mediated
ILV fate decision	ESCRT‐III & VPS4 complex formation	Rab31 inhibits Rab7 to block lysosomal degradation
Common molecules	Flotillins, Rab GTPases	Flotillins, Rab GTPases
Pathway regulation	Rab5, TSG101, Flotillin‐1, Doa4 deubiquitinase	Rab31, EGFR phosphorylation, TBC1D2B,
Disease association	Carcinogenesis, neurodegenerative diseases, dysregulated sorting in viral budding	Mainly studied in neurodegenerative diseases, less in cancer and others
Pathway inhibitors	Inhibition of ESCRT proteins (e.g., VPS, Syntenin, Alix, CHMPs)	nSMase2 inhibitors, HSP70 inhibitors, Rab31/Rab7 modulation
Membrane profile	Enriched in tetraspanins, phospholipases	Enriched in ceramides, HSP70/90, lipid rafts

## 3 MDVs in Mitochondria Quality Control

Mitochondria are dynamic organelles that continuously undergo events of fission and fusion. Much like cells, mitochondria communicate among themselves through a dynamic interconnected network (Horbay and Bilyy 2016). Disruption of this form of communication, such as through the generation of excessive reactive oxygen species (ROS), can lead to mitochondrial damage and trigger the onset of various diseases (Li et al. 2013). In response, mitochondria have several strategies to mitigate damage. (i) A single mitochondrion will attempt to repair itself internally (Horbay and Bilyy 2016). (ii) The damaged mitochondria can directly fuse with healthy counterparts via the unfolded protein response (UPR). During fusion, damaged components are replaced, fused or eliminated. The process involves mitochondrial stress and triggers communication between mitochondria and the nucleus to orchestrate the repair or removal of damaged components. The UPR is a cellular stress response activated by the accumulation of unfolded or misfolded proteins in the endoplasmic reticulum (ER). This response aims to restore ER homeostasis by reducing the protein folding load, increasing protein folding capacity, and promoting degradation of misfolded proteins. If unresolved, the UPR can trigger cell death (Pellegrino et al. 2013). (iii) Formation of MDVs to selectively export damaged or unwanted cargo for degradation (Roberts et al. 2016; Rippstein et al. 2018). (iv) When the damage is beyond repair, mitochondria are selectively degraded through autophagy, a process known as mitophagy (Kim et al. 2007).

MDVs are thus critical for mitochondrial quality control and cellular homeostasis, as abnormalities in MDV biogenesis or cargo composition have been linked to a range disorders and diseases such as autoimmunity, neurodegenerative and cardiovascular diseases (McLelland et al. 2014; Picca et al. 2020; König and McBride 2024; Li et al. 2020).

## 4 Mitophagy, Mitochondrial Fragmentation and MDVs Cross Paths With MVB and EV Release

MDVs are not classically considered as an EV due to their origin and trafficking routes. MDVs typically bud off from mitochondria and are directed towards degradation pathways, resembling the fate of ILVs within MVBs, rather than secretion as EVs (Horbay et al. 2022; Roberts et al. 2016; Soubannier, Rippstein, et al. 2012). However, in certain cases, the mitochondrial content can be released extracellularly. For instance, mature osteoblasts secrete whole mitochondria and MDVs via the CD38/cADPR signalling pathway to promote osteogenesis in recipient cells (Suh et al. 2023). An increase of MDV release is associated with mitochondrial fragmentation and mitophagy, particularly under conditions of mitochondrial dysfunction. This includes disruption of mitochondrial cristae structure due to OPA1 deficiency, *FIS1* overexpression, and formation of mitochondrial donuts (a mitochondrial morphology observed after hypoxia‐reoxygenation) (Suh et al. 2023). Mitophagy is a selective form of macroautophagy that clears dysfunctional mitochondria in post‐mitotic cells, limits the accumulation of ubiquitinated proteins and ROS, and prevents the onset of apoptosis. Similar to autophagy, mitophagy involves the encapsulation of mitochondrial cargo within a double‐membraned vesicle, preventing the accumulation of potentially harmful components (McLelland et al. 2014; Towers et al. 2021). Particularly, OPA1 dysfunction is linked to MDV formation, mitochondrial fission and mitochondrial donut formation (Suh et al. 2023). Impaired clearance of damaged cargo is linked to a number of degenerative diseases, particularly Parkinson's disease where PINK1 and Parkin are involved in MDV formation and cargo packaging (Soubannier, Rippstein, et al. 2012). In this case, PINK1 and Parkin are not involved in the initiation of mitophagy, but rather a MDV‐dependent protein degradation mechanism (McLelland et al. 2014).

**FIGURE 1:**
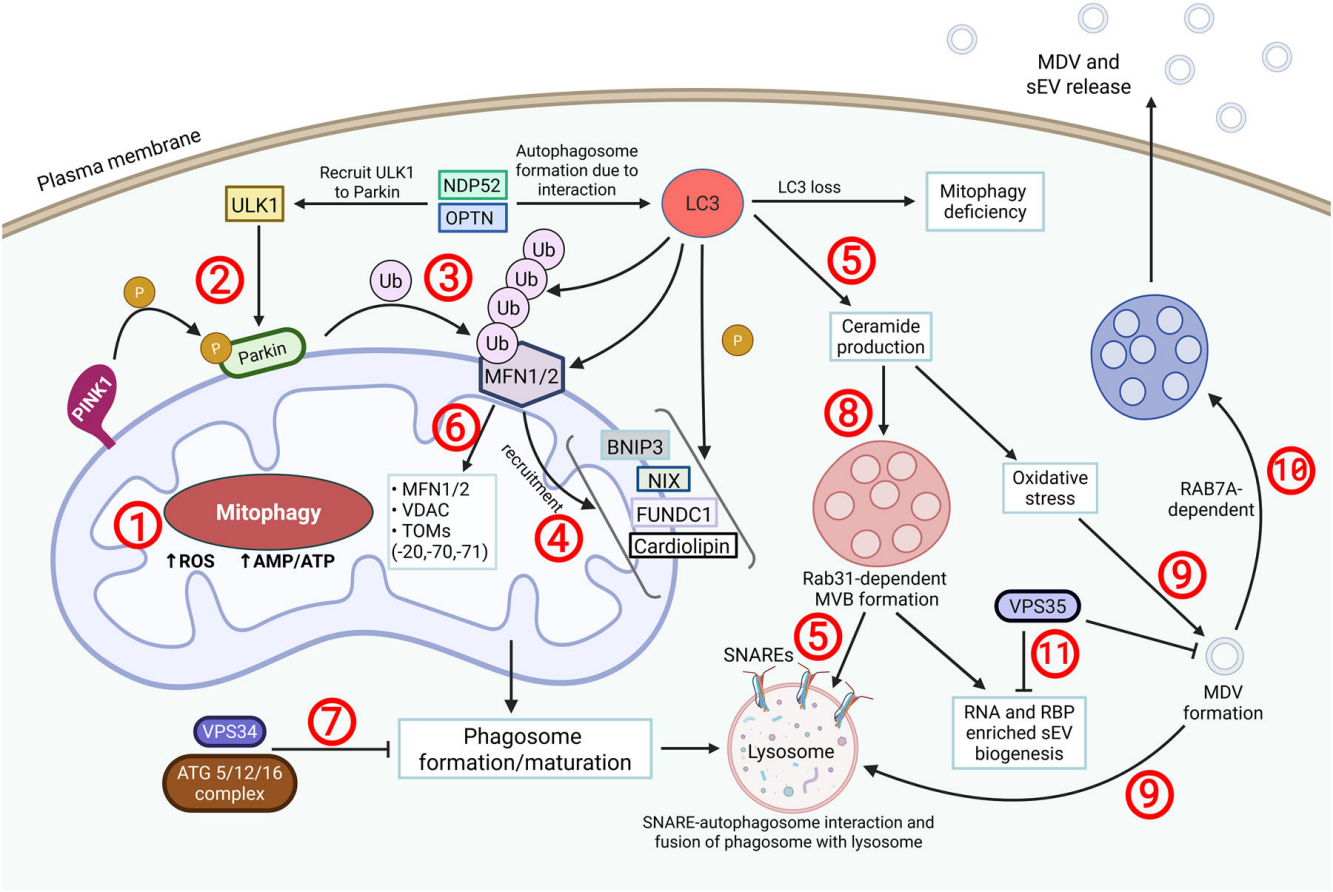
Mitophagy and MDVs maintain mitochondrial dynamics. To prevent excessive ROS accumulation, mitochondria initiate mitophagy following the loss of electrochemical mitochondrial membrane potential (Mashburn and Whiteley 2005). Mitophagy begins with PINK1‐mediated phosphorylation of Parkin (Lötvall et al. 2014) and subsequent MFN1/2 ubiquitination (Soubannier et al. 2012). MFNs recruit BNIP3, NIX, FUNDC1 and Cardiolipin to target damaged mitochondria, while LC3 will phosphorylate these four receptors (Sugiura et al. 2014). LC3 is a key player in mitochondrial quality control by promoting the engulfment of defective or damaged mitochondria into phagosomes, including via the MDV pathway (Yamashita et al. 2016). LC3 activity is regulated by NDP52 and OPTN, which recruit ULK1 to Parkin, enabling further Parkin‐mediated ubiquitination and phosphorylation of MFN1/2 (Lötvall et al. 2014; Soubannier et al. 2012), VDAC and TOM20 (Neuspiel et al. 2008). If not inhibited by the VPS34 and ATG5/12/16 complex, the phagosome is trafficked to the lysosome for degradation (Matheoud et al. 2016). Loss of LC3 results in mitophagy deficiency, impaired ceramide retention, that requires RAB31, will disrupt MVB formation (McLelland et al. 2014), oxidative stress and MDV formation. Most MDVs are degraded in lysosomes or peroxisomes (Théry et al. 2018). However, a subset can be packaged into MVBs and released extracellularly via RAB7‐dependent trafficking of the MDV towards the MVB (Welsh et al. 2024). On the other hand, VPS35 can inhibit both MDV and sEV biogenesis (Horbay et al. 2022).

During mitophagy, cargo encapsulation involves protein ubiquitination mediated by the PINK1/Parkin pathway (Dagda et al. 2009). Mitochondrial depolarization triggers the accumulation of PINK1 on the outer mitochondrial membrane, where it phosphorylates Parkin. PINK1 recruits mitophagy receptors such as NDP52 and OPTN, which in turn recruit ULK1, DFCP1 and WIPI1 (Lazarou et al. 2015). Parkin‐mediated ubiquitination and phosphorylation of outer mitochondrial proteins (such as VDAC, MFN1/2 and TOM20) facilitates the recruitment of adaptor proteins (such as NDP52, OPTN, TAX1BP1 and NBR1). The interaction of these adapter proteins with LC3 leads to the formation of autophagosomes. Proteins, such as VPS34 and the ATG5/12/16 complex, promote autophagosome maturation and closure. These autophagosomes then fuse with lysosomes via SNARE‐containing proteins (Towers et al. 2021; Liu et al. 2012; Safa 2013). In cases of LC3 loss or mitophagy impairment, mitochondria redirect ubiquitinated protein into MDVs for lysosomal degradation (Towers et al. 2021). LC3 activity during receptor‐dependent mitophagy can promote ceramide retention at the MVB during ILV formation, facilitating the selective incorporation of RNA binding proteins and non‐coding RNA into EVs in an ESCRT‐independent manner (Leidal and Debnath 2020). The balance of MDV formation and mitophagy is summarized in Figure 1.

Oxidative stress can alter MDV cargo composition, targeting these vesicles for lysosomal degradation. The addition of lysosome inhibitors stimulates oxidative stress, leading to a 5‐ to 10‐fold increase in MDV accumulation (Soubannier, McLelland, et al. 2012). Oxidative stress induced disruption of lysosome and MVB fusion can also result in EX release (Duan et al. 2024). Moreover, inhibition of oxidative phosphorylation (OXPHOS) by antimycin A also enhances MDV formation, with increased packing of various detrimental cargo, such as ROS, proteins associated with oxidative stress, autophagy/mitophagy related proteins, hyper‐reactive cysteine residues and redox enzymes (Vasam et al. 2021).

The mechanisms whereby MDVs balance life and death are similar to ESCRT‐dependent ILV packaging of ubiquitinated cargo into MVBs. Typically, ILVs are degraded upon MVB‐lysosome fusion, but a subset escapes via MVB fusion with the plasma membrane, releasing EXs (Babst 2011; van den Boorn et al. 2013). From an evolutionary perspective, the biogenesis of MDVs resembles EC formation. Although MDVs and ILVs can colocalize within MVBs, the MDV cargo is also degraded by the peroxisome. These MDVs and ILVs can carry ubiquitinated and SUMOylated proteins that are tagged for degradation (Braschi et al. 2010; Ryan et al. 2020; Kunadt et al. 2015).

In yeast, the mitochondrial import subunits TOM70 and TOM71, which are homologs of mammalian TOM20, are required for the formation of small vesicle‐like structures known as mitochondrial derived compartments (MDCs). Mechanistically, TOM70 and TOM71 recruit the mitochondrial outer membrane protein MFB1, a protein critical for mitochondrial morphology and vesicle trafficking, to MDCs (Galan et al. 2001). Analogous to MDV cargo delivery, MDCs serve as a mechanism for vesicular elimination of mitochondrial waste and certain proteins. MDCs have a wider size range than MDVs, containing inner and outer mitochondrial membrane components (Hughes et al. 2016). Unlike MDV formation and PINK1/Parkin‐mediated autophagy, this mechanism of MDC delivery is believed to be mediated by FIS1 and DRP1 (Hughes et al. 2016).

A study of melanoma derived sEVs revealed enrichment of mitochondrial membrane proteins, termed mitochondrial EVs (Jang et al. 2019). The cargo of these mitochondria EVs included the mitochondrial inner membrane protein MT‐CO2, fragments of the mitochondrial genome, and active mitochondrial enzymes. This finding indicates that mitochondrial EVs may serve as biomarkers for diseases, such as cancer.

Unlike mitophagy, MDVs can emerge rapidly in response to stressors such as accumulated ROS and OXPHOS inhibition (Sugiura et al. 2014; Vasam et al. 2021). In *Drosophila melanogaster* brain cells, PINK1 or Parkin mutations not only promote mitophagy but also impair mitochondrial protein turnover, linking these mutations to MDV formation (Vincow et al. 2013). This might be the first evidence linking MDV formation to PINK1 and Parkin mutations. Proteolysis of unfolded, unassembled, oxidized, ubiquitinated or misfolded mitochondrial proteins and lipids is essential for mitochondria function. As organelles that selectively packages cargo into MDVs for degradation, mitochondria are important for tagging MDVs for proper trafficking of unwanted cargo. Failure to remove the damaged cargo MDVs is implicated in degenerative diseases, particularly those involving PINK1 and Parkin (Soubannier, Rippstein, et al. 2012). In this case, PINK1 and Parkin are not involved in mitophagy launch, but rather fostering a MDV‐dependent protein degradation mechanism (McLelland et al. 2014). An analogous vesicle shedding mechanism of MDV formation exists in α‐proteobacteria, the evolutionary ancestors of mitochondria. This vesicular shedding mechanism plays an important role in bacteria survival that parallel the role of MDVs in eukaryotic cells, such as transportation of biologically active proteins, supply of nutrients, support biofilm formation and promoting pathogenicity (Kulp and Kuehn 2010).

## 5 Types of MDVs and Their Content

**FIGURE 2:**
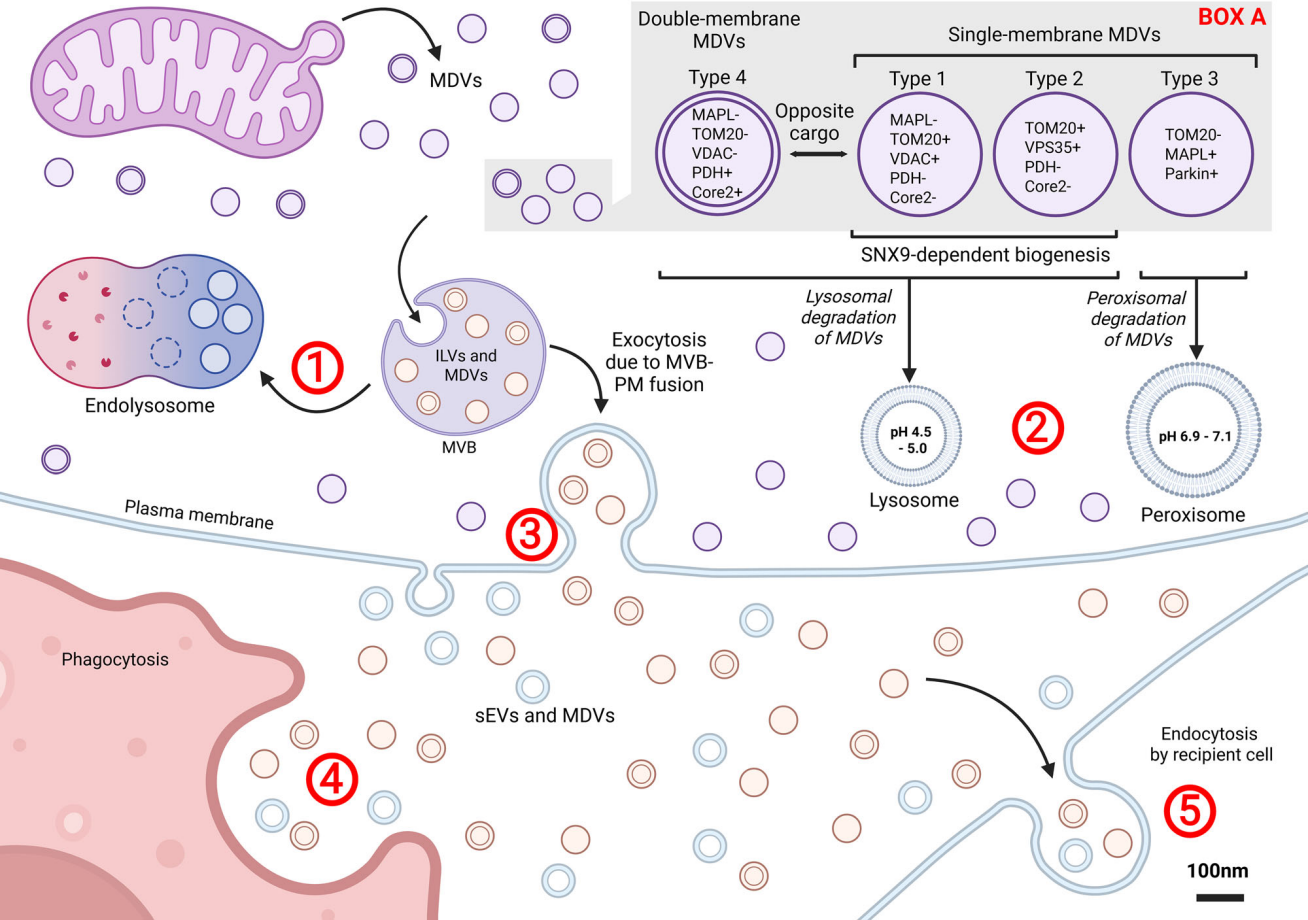
Types of MDVs. There are four main types of MDVs that are classified in accordance with cargo content and membrane structure. Types 1–3 are single‐membrane while Type 4 is double‐membrane (Box A). Depending on the cargo content, MDVs are directed to lysosomes or peroxisomes for cargo degradation. SNX9 is involved in the biogenesis of Type 1 and Type 2 MDVs. The classification is based on the presence or absence of specific proteins: Core2, MAPL, PDH, TOM20, VDAC and VPS35. MDVs may enter endosomes, which later fuse with lysosomes for degradation (Mashburn and Whiteley 2005). Alternatively, MDVs can be incorporated into MVBs and degraded by lysosomes or peroxisomes (Lötvall et al. 2014) or escape the cell in an EV‐like manner via plasma membrane‐MVB fusion (Soubannier et al. 2012). In this case, both MDVs and sEVs can be endocytosed/phagocytosed by recipient cells such as antigenpresenting cells (Sugiura et al. 2014; Yamashita et al. 2016).

MDVs carry mitochondrial proteins and lipids to other organelles, depending on their origin and cargo selection—a process that is highly regulated and non‐random (Soubannier, McLelland, et al. 2012; Soubannier, Rippstein, et al. 2012). MDVs exhibit diverse cargo content and morphologies (Figure 2). Given that mitochondria are double‐membrane organelles, MDVs were initially proposed to be double‐membrane structures with a uniform diameter, analogous to cellular membranes (Park et al. 2001). Some MDVs contain both inner and outer mitochondrial membrane components, while others consist solely of the inner membrane. This demonstrates that MDVs comprise a heterogeneous and highly selective pathway utilized for mitochondrial quality control, with single‐membrane MDVs more frequently observed. These two types of MDVs differ in electron density between the inner membrane and intermembrane space (Soubannier, Rippstein, et al. 2012). Due to the heterogeneous nature of MDV cargo, each vesicle is trafficked via specific routes involving mitochondrial‐anchored protein ligases, often targeting delivery to the peroxisome (Neuspiel et al. 2008). Four major types of MDVs have been identified based on cargo content and number of lipid layers: (Mashburn and Whiteley 2005) single membrane TOM20‐positive (TOM20^+^), (Lötvall et al. 2014) single membrane TOM20^+^ and VPS35‐positive (VPS35^+^), (Soubannier, McLelland, et al. 2012) single membrane MAPL‐positive (MAPL^+^) and (Sugiura et al. 2014) double‐membrane pyruvate dehydrogenase‐positive (PDH^+^) or Core2‐positive MDVs (Core2^+^) (Sugiura et al. 2014; Neuspiel et al. 2008; Soubannier, Rippstein, et al. 2012).

In Types 1 and 2 MDVs, knockdown of SNX9, a protein essential for clathrin‐mediated endocytosis and PDH^+^ MDVs biogenesis, reduces TOM20^+^/PDH^−^ MDVs in autophagy‐deficient cells (Towers et al. 2021). Although MDV numbers increase in SNX9‐deficient cells, their delivery to lysosomes is impaired, suggesting SNX9 may function similarly to autophagy adaptors in trafficking MDVs and regulating mitochondrial antigen presentation (MitAp) (Matheoud et al. 2016).

Type 3 MDVs carry the mitochondrial outer membrane protein, MAPL. MAPL is a mitochondrial SUMO E3 ligase involved in mitochondrial fission and cargo‐specific recruitment of peroxisomes (Neuspiel et al. 2008). These MDVs also contain Parkin, but lack TOM20. The absence of MAPL in MDVs leads to lysosomal delivery of oxidized protein cargo (Rippstein et al. 2018).

Type 4 MDVs are double‐membrane vesicles that contain PDH or Core2 E2/E3 subunit proteins but lack TOM20 and VDAC. These MDVs are formed via mitochondria complex III inhibition (e.g., Antimycin A treatment in COS7 cells) independent of mitophagy, involving PINK1 and Parkin recruitment to the mitochondrial membrane (Sugiura et al. 2014; Soubannier, Rippstein, et al. 2012). A similar class of double membrane vesicles has been described, called mitovesicles, however they are distinct from MDVs (Box 1).

**FIGURE 3:**
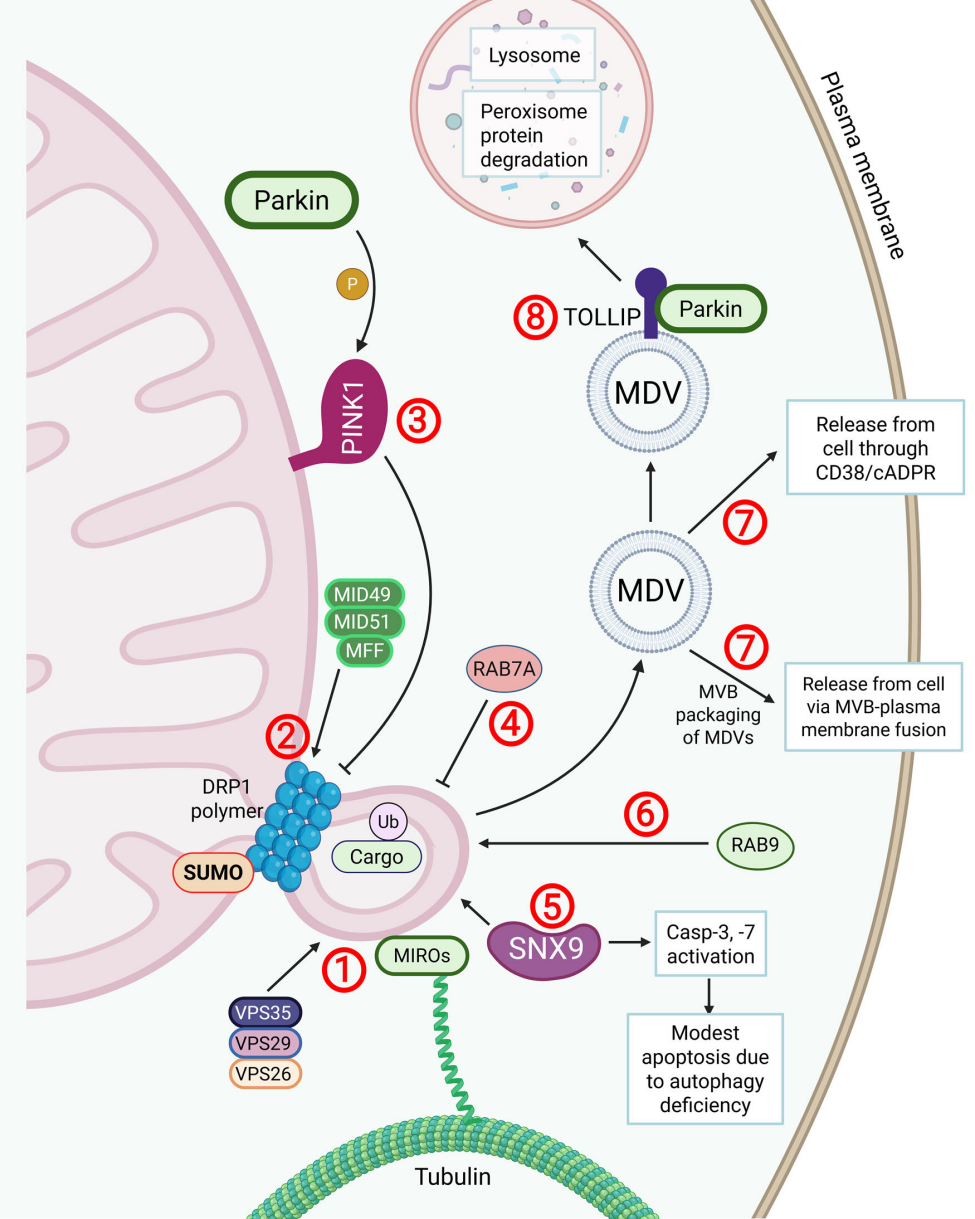
The role of SNX9 and DRP‐1 in MDV release. MDV formation begins with membrane curvature initiation where VPS35, a core component of the VPS35/VPS29/VPS26 retromer complex, plays a key role, and MIRO proteins guide tubulation (Mashburn and Whiteley 2005). MID49, MID51 and MFF proteins direct DRP1, a mitochondrial fission protein involved in mitochondrial biogenesis, to polymerize and form a neck around the budding MDV (Lötvall et al. 2014). RAB7A and PINK1/Parkin can inhibit DRP1‐dependent MDV formation (Soubannier et al. 2012; Sugiura et al. 2014). SNX9 acts as an adaptor protein essential for MDV formation, lysosomal delivery of MDVs, mitochondrial cargo packaging into sEVs and caspase activation (Yamashita et al. 2016). In contrast, RAB9 promotes DRP1‐mediated MDV release (Neuspiel et al. 2008). Once formed, MDVs can be released from the cell via MVBs or through a CD38/cADPR dependent process (Matheoud et al. 2016), though most are directed to lysosomes or peroxisomes for cargo degradation. Tollip coordinates the trafficking of damaged cargo into MDVs for degradation (McLelland et al. 2014).

Recent studies have described a DRP1‐dependent mechanism of MDV biogenesis (Figure 3) (Towers et al. 2021; König et al. 2021). During MDV formation, DRP1 polymerizes around the vesicle neck of the future MDV—this facilitates scission and release of the vesicle from the mitochondrion (Towers et al. 2021). Deficiency of a protein involved in membrane curvature, SNX9, is associated with loss of ATG7 and reduction of MDV formation, indicating that autophagy‐deficient cells rely on MDV‐mediated clearance of severely damaged mitochondrial cargo via the lysosome. A later study demonstrated that MDVs can be generated through classical mitochondrial fission proteins, such as MIRO1/2 (König and McBride 2024; König et al. 2021). The MIRO proteins pull and extend the curvature due to their interaction with Tubulin and the MID proteins (MID49 and MID51) and MFF, with DRP1 executing final scission (König et al. 2021). This highlights the flexibility of mitochondrial MDV formation across multiple pathways.

MDV membrane scission involving DRP1 begins with VPS35, a component of the retromer VPS29/VPS35/VPS26 complex involved in protein packaging and cargo sorting, which initiates membrane curvature of the future MDV (König et al. 2021). VPS35 facilitates MAPL and MDV transport to peroxisomes and regulates mitochondrial membrane curvature during MDV formation (Braschi et al. 2010). VPS35 interacts with RAB7A to stabilize cargo‐selective complexes and recruit the retromer complex to late endosomes (Priya et al. 2015). Loss of VPS35 leads to intracellular accumulation of TOLLIP, a negative regulator of TOM20⁺ MDV formation, and disrupts Parkin‐dependent endosomal trafficking (Ryan et al. 2020). The loss of VPS35 also reduces EV release, opposing the function of RAB11 (Walsh et al. 2021), a protein involved in the transport of recycling endosomes and MVBs to the plasma membrane, as well as in the EX fusion with the plasma membrane (Marie et al. 2023). Thus, VPS35 may act as a molecular switch between MVB‐ and MDV‐mediated protein degradation pathways, with VPS35 loss impairing both EV and MDV biogenesis.

**BOX 1 | Mitovesicles**

A novel class of double‐membrane EVs derived from mitochondria has been described, termed mitovesicles (mitoEVs) (D'Acunzo et al. 2021). These EVs contained multiple mitochondrial proteins that are distinct from MVs or EXs. Like sEVs, mitoEVs deliver mitochondrial components that affect the function of recipient cells. Under pathological conditions, mitoEVs can deliver functional mitochondrial fragments to recipient cells, helping to restore normal mitochondria dynamics and function (Lin et al. 2015). Mostly discovered in the brain tissue, these EVs have been described to affect synaptic function and are strongly associated with multiple neurological conditions. Particularly, mitoEVs isolated from a mouse model of Down syndrome can reduce long‐term potentiation of hippocampi (D'Acunzo et al. 2024). Interestingly, tissue‐derived mitoEVs can contain functional mitochondria that lead to increased mitochondrial biogenesis and reduced mitochondrial damage in recipient cells. These mitoEVs attenuated different types of tissue injuries by rescuing mitochondrial injury and reducing bystander inflammation (Lou et al. 2025). MitoEVs also harbour a variety of mitochondria‐derived material, such as mtDNA, mtRNA, ATP and intact mitochondria (Wang et al. 2020; Rabas et al. 2021; Falchi et al. 2013). Despite their mitochondrial origin, mitoEVs are not considered MDVs due to key differences in their biogenesis and function: (i) MDVs originate from the mitochondria whereas mitoEVs are released from the plasma membrane; (ii) mitoEVs are delivered to the plasma membrane through MVB fusion, enabling extracellular release; (iii) mitoEVs carry functional mitochondrial components, while MDVs typically transport damaged proteins destined for degradation and (iv) unlike MDVs, mitoEVs do not fuse with lysosomes or peroxisomes due to their release pathway. The distinction between MDVs and mitoEVs remains challenging due to overlapping features and difficulties in extraction and purification. Recently, a protocol involving filtration, sequential centrifugation and iodixanol‐based density gradient was developed for the extraction of mitoEVs from the brain, which was more efficient and reproducible than the traditional sucrose gradient protocol (D'Acunzo et al. 2022). Nonetheless, their differences in origin, cargo and trafficking pathways underscore the need for careful classification in EV research.

## 6 A Comparative Analysis of MDVs and sEVs

sEVs and MDVs share several key features, particularly roles in cargo degradation, evolutionary origin via the endosymbiotic theory, involvement of the ESCRT machinery in cargo packaging, and overlapping physical characteristics such as size and morphology. However, important distinctions exist between the two classes of vesicles. Unlike EXs, the ability of MDVs to exit the cell is contentious. If MDVs are secreted, their size may preclude incorporation into ILVs, potentially limiting their selective uptake by recipient cells. While MDVs could potentially be incorporated into larger vesicle types such as MVs (size range of 30–500 nm) or ApBs (up to 1 µm), these pathways are generally associated with non‐specific uptake and recycling via pinocytosis, rather than targeted delivery (Schwager and Reinhart‐King 2020; Atkin‐Smith and Poon 2017). Interestingly, cells may redirect proinflammatory mitochondrial content from MDVs into EVs under certain conditions (Todkar et al. 2021). This suggests that MDV‐specific proteins may participate in packaging mitochondrial cargo into EVs.

MDVs share similarities with cargo packaging and delivery with sEVs. Some MDV cargo may escape lysosomal degradation and be released extracellularly via MVB fusion with the plasma membrane (Tucher et al. 2018; Moreno‐Gonzalo et al. 2018). MDVs have been observed within MVBs, and their DNA and damaged protein content resemble the selective cargo incorporation seen in EXs (Roberts et al. 2016; Soubannier, Rippstein, et al. 2012; Braschi et al. 2010). MDVs also share similarities to ECs, which, like MDVs, are similar in size and are generated via a direct membrane budding mechanism. However, MDV cargo is often ubiquitinated and tagged for degradation, akin to ILVs. From an evolutionary perspective, MDVs may be considered downsized analogues of ApBs, retained within the cell for recycling and reuse, rather than being released (Wickman et al. 2012). Furthermore, while EXs require MVB formation and release into the extracellular space, MDVs can be directly released, a trait they share with MVBs and ApBs (Babst 2011; Roberts et al. 2016; Simons and Raposo 2009). Thus, MDVs may represent a distinct vesicle class that blends features of both EXs and ApBs.

Both MDVs and sEVs share membrane surface molecules with mitochondria and the plasma membrane. Both MDVs and sEVs are enriched with specific heat shock proteins, tetraspanins and other specific surface markers (Lötvall et al. 2014). However, MDVs uniquely contain mitochondrial‐specific proteins such as TOMs, TIMs, VSP35, MAPL, PDH and Core2 E2/E3 subunit protein (Lötvall et al. 2014; Sugiura et al. 2014; Neuspiel et al. 2008; Roberts et al. 2016; Braschi et al. 2010). Notably, TOM20 is commonly used as a negative EV marker in sEV purification as its presence indicates potential mitochondrial contamination (Théry et al. 2018).

A distinct feature between sEVs and MDVs is that sEVs are actively released for intercellular communication, whereas MDVs typically remain intracellular. sEVs carry a broad range of cargo, including nucleic acids such as miRNAs (Tran 2016). In contrast, MDVs primarily contain material destined for degradation in peroxisomes and lysosomes (Soubannier, McLelland, et al. 2012; Neuspiel et al. 2008; Rippstein et al. 2018; Todkar et al. 2021). Nonetheless, sEVs have been shown to carry mitochondrial‐derived material such as mitochondrial DNA (mtDNA) (Zhang et al. 2012), blurring the lines between these vesicle types. As MDVs can be released in an SNX9‐dependent manner (Zecchini et al. 2023), this raises the possibility that mitochondria may package exosomal or other sEV cargo into MDVs. This highlights the need for further investigation into the interplay between mitochondrial vesicle formation and EV biology.

### 6.1 Similarities Between sEVs and MDVs

MDVs and sEVs share several mechanistic and structural similarities, particularly in their use of the ESCRT pathway for cargo sorting and delivery to the lysosome for degradation. Both MDVs and sEVs types rely on the formation of SNARE complexes to mediate membrane fusion events. There are two classifications of SNARE complexes: v‐SNAREs that are located on vesicle membranes, facilitating fusion with target membranes and t‐SNAREs that are located on terminal membranes, enabling docking and fusion (Yoon and Munson 2018). Multiple SNARE proteins, such as Syntaxin‐2/4/3/18, SNAP‐23/29 and VAMP‐7, are essential for vesicle‐membrane fusion and sEV secretion (Liu et al. 2023; Hessvik et al. 2023). Among these, SNAP‐29 seems to be critical as removal of SNAP‐29 caused the greatest reduction in EV release, especially sEVs enriched with CD63 and the ESCRT‐proteins, Syntenin‐1 and TSG‐101 (Hessvik et al. 2023). However, levels of Annexin A2 levels (a classical EC biomarker) and LC3B and p62 (secretory autophagy proteins) remained unchanged, suggesting a specific role for SNAP‐29 in sEV biogenesis. In MDVs, Syntaxin‐17, a core mitochondrial SNARE, is essential for delivering PINK1/Parkin‐dependent MDVs to lysosomes under stress conditions (McLelland et al. 2016). Syntaxin‐17 forms a ternary SNARE complex with SNAP‐29 and VAMP‐7, linking MDVs to late endosomes and lysosomes. These shared SNARE components underscore a mechanistic overlap between sEV secretion and MDV‐lysosome fusion.

Another shared factor is SNX9, a protein involved in membrane curvature at both the mitochondrial and plasma membranes. SNX9 is critical for vesicle biogenesis and targeted packaging of mitochondrial proteins into EVs. Also, SNX9 is involved in membrane curvature of both plasma membrane and mitochondrial membrane (Todkar et al. 2021). SNX9 directly stimulates the GTPase activity of Dynamin‐1 and Dynamin‐2, two essential components in vesicle formation (Towers et al. 2021; van der Bliek et al. 1993). GTPase activation potentiates assembly‐stimulated GTPase activity on liposomes and SNX9 then co‐localizes with clathrin‐coated pits during the late stage of vesicle formation (Soulet et al. 2005). SNX9 together with Dynamin‐2 play a critical role in clathrin‐mediated endocytosis (Kaksonen and Roux 2018). Also involved are RAB9 and RAB7, which are small GTPases that regulate the activity and trafficking of late endosomes/lysosomes (Bento et al. 2013). RAB9 and SNX9 regulate MDV formation and budding from mitochondria, whereas RAB7 mediates membrane fusion between MDVs and endosomes (Matheoud et al. 2016). LKB1‐mediated mitochondrial recruitment of RAB9 promotes MDV shedding (Zhao et al. 2024) and RAB7 deletion increases EV release containing ubiquitinated cargo and intact mitochondria (Liang et al. 2023). Notably, SNX9 regulates the formation of inner membrane/matrix MDVs, but not outer membrane MDVs. Another study has shown that MDVs are essential for the release of mitochondrial proteins in EVs in macrophages. Upon mitochondrial damage, this release mechanism is blocked to prevent the release of pro‐inflammatory damaged mitochondrial cargo (Todkar et al. 2021). In this context, the sEV fraction is enriched with EX positive markers but also contains high levels of TOM20 and low levels of PDH and cytochrome C. This indicates that MDVs are capable of escaping the cell under normal conditions (Todkar et al. 2021).

A further ancestral link between MDVs and EVs is the shared presence of heat shock proteins, such as HSP70 (Andre et al. 2004). Mitochondrial mtHSP70 facilitates the import of proteins into the mitochondrial matrix via TIM17/23, a function similar to the EV‐associated HSP70 (Ting et al. 2017). As HSP70 is a classical EV biomarker, the presence of mtHSP70 supports the notion that mitochondria are capable of generating EVs.

### 6.2 MVBs, MDVs and Protein Degradation via Peroxisome and Lysosome

There are two known destinations for MDV cargo: the lysosome and peroxisome. Single‐membraned MDVs are more commonly trafficked to peroxisomes or lysosomes, while double‐membrane Type 4 MDVs are proposed to be destined for lysosome degradation (Roberts et al. 2016). The connection between MDVs and peroxisomes is particularly notable due to the peroxisome's role in oxidative metabolism, ROS detoxification and cellular signalling (Waterham et al. 2007; Bonekamp et al. 2009). MDVs function as vesicle‐like bodies that transport unwanted mitochondrial cargo to peroxisomes, functioning similarly to EXs and MVs (Neuspiel et al. 2008; Roberts et al. 2016; Rippstein et al. 2018; Soubannier, Rippstein, et al. 2012). Interestingly, the ESCRT‐III complex, which is essential for EX release, is required for peroxisome biogenesis. ESCRT‐III components such as VPS20 and SNF7 (mammalian CHMP4) regulate the final scission of preperoxisomal vesicles, which mature into functional peroxisomes (Mast et al. 2018). This shared machinery suggest that peroxisomes may be evolutionary related to MVBs, further linking MDV trafficking to broader vesicular transport systems.

### 6.3 The Endosymbiotic Theory and Bacteria‐Derived EVs

The endosymbiotic theory posits that mitochondria originated from ancestral α‐proteobacteria that entered into a symbiotic relationship with early eukaryotic cells (Gould et al. 2016; Margulis, 1981). Bacteria are well known for using EVs to exchange signalling molecules to facilitate gene expression and quorum sensing (Mashburn and Whiteley 2005). These vesicles also have roles in host invasion, inter‐bacterial competition and biofilm formation under stress conditions (Deatherage and Cookson 2012).

MDVs share several features with bacterial EVs, including single/double‐membranes, cargo transportation and immune modulation (Neuspiel et al. 2008; Roberts et al. 2016; Tiku and Tan 2021). Mitochondria, like bacteria, exhibit autonomous behaviours such as DNA replication, programmed cell death and selective cargo elimination via lysosomal and peroxisomal recycling (Horbay and Bilyy 2016; Towers et al. 2021; Dagda et al. 2009). Both mitochondria and cells can encapsulate cargo in lipid bilayer vesicles, further supporting their shared evolutionary lineage. A novel aspect of this symbiosis is the ability of sEVs and MDVs to be encapsulated within MVBs, protecting these vesicles from endocytosis‐mediated degradation and enabling controlled secretion (Colombo et al. 2014).

Bacteria and archaea produce bacteria membrane vesicles (BMVs). BMVs are primitive structures ranging from 20 to 500 nm—similar in size to MDVs, which themselves range 50–150 nm (Toyofuku et al. 2019). BMVs selectively package proteins, nucleic acids, lipids, metabolites and toxins (Nagakubo et al. 2020). There are several outcomes from shedding of BMV cargo: horizontal gene transfer (e.g., plasmids), transport of virulence factors (e.g., bacteriophages, eukaryotic host defence factors), export of cellular metabolites (e.g., antibiotics, toxins) and detoxification. Consequently, BMV secretion constitutes a universal mechanism of interaction and communication between prokaryotic and eukaryotic organisms (Chronopoulos and Kalluri 2020). BMVs can also act as decoys, triggering immune detection and phagocytosis while allowing bacterial populations to expand (Pathirana and Kaparakis‐Liaskos 2016). Mitochondria mirror these features by shuttling metabolites to ER, acquiring lipid building blocks for its biogenesis, and eliminating waste via mitophagy and MDV trafficking (Shiao et al. 1995). MDV formation can be recognized as a defence mechanism, complementing mitophagy and maintaining mitochondrial integrity (Neuspiel et al. 2008). Collectively, BMVs provide additional evidence for the ancestral link between mitochondrial and bacteria, reinforcing the endosymbiotic theory of mitochondrial origin and the shared capacity for EV generation across eukaryotic and prokaryotic life.

Table 2 below summarizes the main highlights of vesicle characteristics between MDVs and sEVs.

**TABLE 2:** Summary comparison of MDV and sEV characteristics.

Feature	Mitochondria‐derived vesicles (MDVs)	Small extracellular vesicles (sEVs)
Origin	Bud from mitochondria (Sugiura et al. 2014; Neuspiel et al. 2008; Todkar et al. 2021)	Derived from multivesicular bodies (MVBs) or plasma membrane (Lötvall et al. 2014; Colombo et al. 2014)
Size	∼70–150 nm (varies by cell type and condition) (Sugiura et al. 2014; Neuspiel et al. 2008; Todkar et al. 2021)	∼30–150 nm (Lötvall et al. 2014; Colombo et al. 2014)
Membrane structure	Single or less double‐membrane (Sugiura et al. 2014)	Single and double‐membrane (Welsh et al. 2024)
Biogenesis pathway	Mitochondrial membrane budding; Parkin/PINK1‐ or SNX9‐dependent (Lötvall et al. 2014; Neuspiel et al. 2008; Matheoud et al. 2016)	ESCRT‐dependent or ESCRT‐independent pathways (Suo et al. 2024)
Stimuli for release	Oxidative stress, mitochondrial dysfunction, infection (Soubannier, McLelland, et al. 2012; McLelland and Fon 2018)	Cellular stress, hypoxia, oncogenic activation (Kalluri and LeBleu 2020; Robbins and Morelli 2014)
Major cargo	Mitochondrial proteins, lipids, mtDNA, oxidized cargo, OXPHOS components, TOMs (Sugiura et al. 2014; McLelland et al. 2016)	Cytosolic proteins, miRNAs, lipids, surface markers (tetraspanins, Syntenin, Alix, Rab31, other) (Lötvall et al. 2014; Horbay et al. 2022; Wei et al. 2021)
Function	Mitochondrial quality control, antigen presentation, inter‐organelle communication (Soubannier, McLelland, et al. 2012; Matheoud et al. 2016; König and McBride 2024)	Cell‐to‐cell communication, immune modulation, cargo delivery (Simons and Raposo 2009; Robbins and Morelli 2014)
Fate	Delivered to lysosomes, MVB, peroxisomes or secreted under stress (Rippstein et al. 2018; König and McBride 2024; Ryan et al. 2020)	Released into extracellular space; internalized by recipient cells (Simons and Raposo 2009; Robbins and Morelli 2014; Horibe et al. 2018)
Release into extracellular space	Rare, but occurs under inflammatory or pathological conditions (Sugiura et al. 2014)	Common; release is constitutive or regulated (Simons and Raposo 2009, Robbins and Morelli 2014)
Markers	TOM20, MAPL, Core2, PDH, VPS35 (Sugiura et al. 2014; Roberts et al. 2016; Soubannier, Rippstein, et al. 2012)	Tetraspanins (e.g., CD9, CD63, CD81), Alix, Syntenin, HSP70, TSG101 and other (Lötvall et al. 2014; Horbay et al. 2022; Stoorvogel 2015; Fan et al. 2019)
Roles in immunity	Present mitochondrial DAMPs; trigger innate immunity (Matheoud et al. 2016; Deus et al. 2022)	Present antigens, carry immune‐regulatory molecules and RNAs (Greening et al. 2015)
Disease associations	Neurodegeneration (e.g., Parkinson's), inflammation, infection (McLelland et al. 2014; Abuaita et al. 2018)	Cancer, cardiovascular disease, neurodegeneration, autoimmune disorders (Greening et al. 2015; Wu et al. 2020; Rastogi et al. 2021)

## 7 Role of EVs and MDVs in Regulating the Immune Response

**FIGURE 4:**
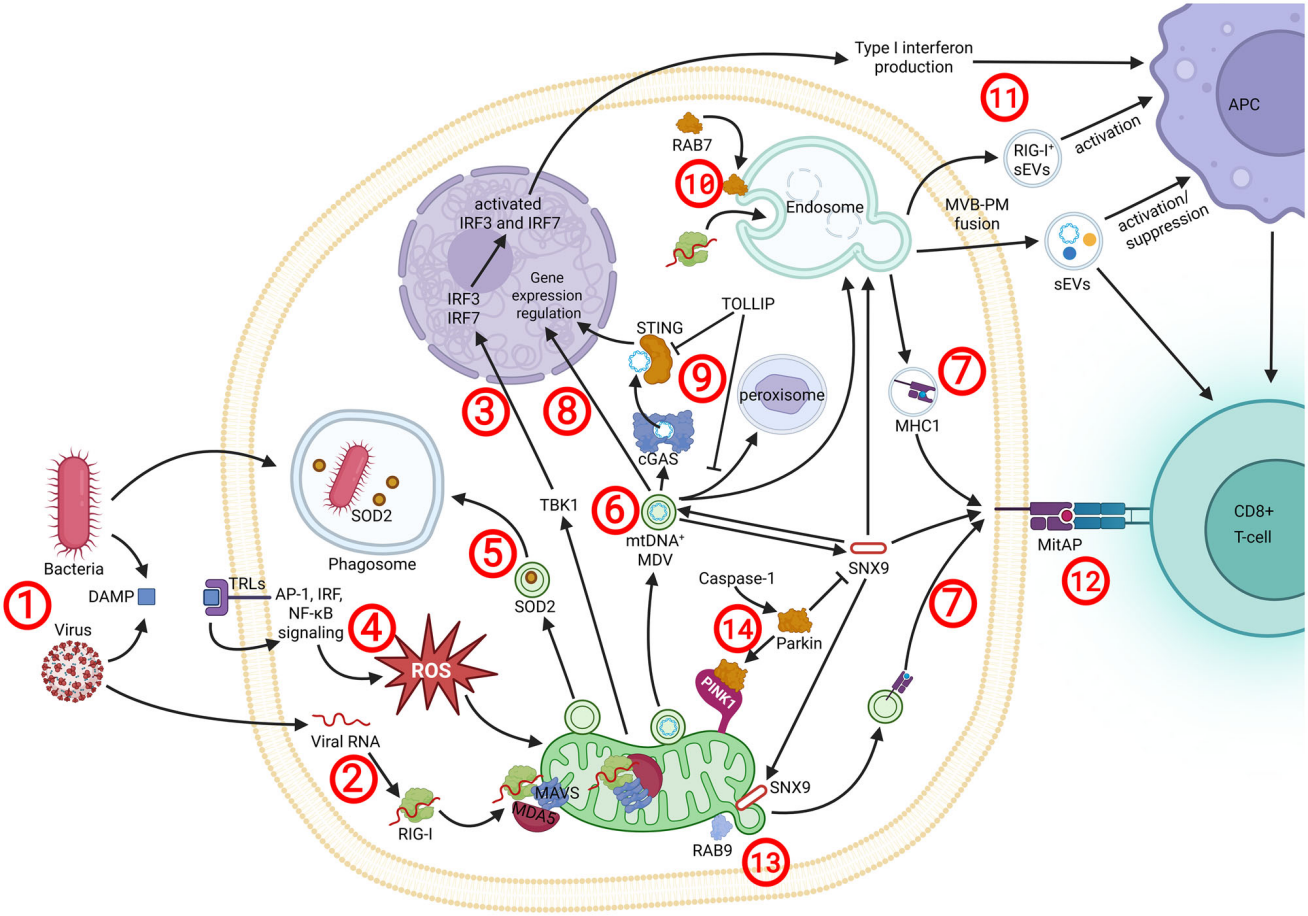
The role of sEVs and MDVs in immunity. Bacteria and viruses can trigger immune responses via MDVs and sEVs (Mashburn and Whiteley 2005). Viral RNA activates RIG‐I, initiating the RIGI‐MDA5‐MAVS complex (Lötvall et al. 2014), which activates TBK1 and leads to Type I IFN production via to IRF3/7 activation (Soubannier et al. 2012). Bacterial and viral DAMPs activate TLRs, leading to ROS accumulation (Sugiura et al. 2014) and MDV release. These MDVs could carry bacteria‐targeting SOD2 (Yamashita et al. 2016), mtDNA (Neuspiel et al. 2008) and MHC‐I molecules (Matheoud et al. 2016). mtDNA‐containing MDVs can regulate gene expression (McLelland et al. 2014), initiate immune responses through cGAS‐STING pathway (Théry et al. 2018), or be degraded via peroxisomes (Théry et al. 2018). The last two events can be inhibited by TOLLIP (Théry et al. 2018). Sensor proteins, such as RIG‐I, is packaged into sEVs in a RAB7‐dependent manner (Welsh et al. 2024) and these sEVs can activate antigen presenting cells and modulate T cell responses (Horbay et al. 2022). T cells can also be activated via MitAP (Meldolesi 2018). Proteins involved in EV biogenesis, such as RAB9 and SNX9, facilitate MDV release and antigen presentation to T cells (Welsh et al. 2024). Notably, SNX9, which is essential for MDV release and MitAP, can be inhibited by Parkin and PINK1. To reduce mitophagy, Parkin can be inactivated via Caspase‐1 cleavage (Larios et al. 2020).

EVs have a pivotal role in the complex interplay between innate and adaptive immune cells (Yan and Jiang 2020). Mitochondria and MDVs contribute to immune regulation in ways that parallel sEVs (Matheoud et al. 2016; McLelland and Fon 2018), providing support to the concept that vesicles are central mediators and triggers of immune signalling. Under homeostatic conditions, cells package mitochondrial proteins into EVs to prevent the release of mitochondria‐generated damage‐associated molecular patterns (DAMPs) from being released and thereby causing inflammation (Todkar et al. 2021). However, when mitochondria are damaged or exposed to proinflammatory stimuli, they can release mtDNA, peptides and other mitochondrial components that trigger immune responses (He et al. 2019). One mechanism for immune regulation via mitochondria is MITochondrial Antigen Presentation (MitAP), a process dependent on MDVs that eliminates hazardous cargo while regulating immune signalling (Matheoud et al. 2016). Additionally, mitochondria can be released in EVs and induce immune responses, further complicating our understanding the role and effect of mitochondria in vesicle biogenesis and release (Puhm et al. 2019). In this section, we will highlight how sEVs and MDVs contribute to immune regulation and how their functional pathways intersect (Figure 4).

### 7.1 sEVs and the Immune Response

sEVs carry signalling molecules that can promote or suppress immune responses. Exposure to DAMPs or pathogen‐associated molecular patterns (PAMPs) induces a proinflammatory state in innate immune cells such as macrophages, thereby enhancing immunosurveillance. In a similar fashion, bacteria release BMVs that are detected by immune cells, facilitating host‐pathogen interactions. Pathogen‐derived EVs often contain PAMPs and other pattern recognition receptors, such as RNA and cGAS, which activate innate immune responses (Gangadaran et al. 2023). These interactions can lead to the production of sEVs containing proinflammatory cytokines, such as GM‐CSF, TNFα, IL‐6, IL‐1α and IL‐1β, that recruit and activate additional immune cells from both of the innate and adaptive arms of the immune system (McDonald et al. 2014; Essandoh et al. 2015).

sEVs facilitate antigen transfer from donor cells to antigen presenting cells such as dendritic cells (DCs) and macrophages (van Dommelen et al. 2016), a critical step in initiating adaptive immunity. In this case, attention should be paid to the analysis of the different content and potential immunogenic effect of these sEVs (Kalluri and McAndrews 2023). The immunogenic potential of sEVs depends on their cargo, including immunostimulatory molecules such as RIG‐I. The packaging of RIG‐I into sEVs and trigger type I interferon (IFN) production, stimulates natural killer cell‐mediated cytotoxicity, and activate both DCs and T cells (Daßler‐Plenker et al. 2016; Heidegger et al. 2023; Bek et al. 2019). Moreover, packaging of RIG‐I or STING into sEVs has been shown to enhance immune activation, including the generation of tumour antigen‐specific CD8 T cells (Heidegger et al. 2023; McAndrews et al. 2021). This process is further regulated by autocrine interferon signalling and RAB27A, which are collectively required for the biogenesis and release of immunostimulatory EVs. Such reprogramming of EVs have significant implications for the development of personalized T cell‐based immunotherapies (Heidegger et al. 2023).

### 7.2 MDV‐Mediated Immune Responses

Mitochondria are one of the key regulators of the innate immune response, particularly in response to DAMPs and PAMPs. Particularly, DAMPs can trigger the release of sEVs. One key mitochondrial DAMP is mtDNA, which activates the TLR9 pathway that thereby triggers NF‐κB signalling. Activation of NF‐κB signalling leads to expression of inflammatory mediators, including pro‐oxidant enzymes that ultimately produce high levels of ROS, such as H_2_O_2_ (Zhang et al. 2010). The increased levels of ROS lead to their accumulation within phagosomes via MDVs in a process critically dependent on Parkin, which facilitates both H_2_O_2_ accumulation and bactericidal activity (Sugiura et al. 2014; Abuaita et al. 2018).

MDVs can carry proinflammatory molecules linked to innate immune responses and antimicrobial activity. Double‐membrane bound, low‐density content TOM20‐negative and PHD‐positive MDVs have been observed in fumarate hydratase double knockout mouse kidney cells, resembling MDVs formed via SNX9‐dependent mechanisms (Zecchini et al. 2023; Sugiura et al. 2017). Elevated intracellular fumarate can trigger mtDNA release into the cytosol through SNX9‐mediated MDV formation, activating cGAS–STING and RIG‐I pathways and promoting innate immune gene expression (Zecchini et al. 2023). In a recent study, it was revealed that beta‐hydroxybutyrate improved mitochondrial function via MDVs. Beta‐hydroxybutyrate drove specific lysine beta‐hydroxybutyration of SNX9, and increased SNX9 interaction with the inner mitochondrial membrane and promoted MDV production. These MDVs provided protection from liver‐induced injury and maintained a healthy mitochondrial state (Tang et al. 2025). Interestingly, SNX9, a key player in vesicle trafficking and clathrin‐mediated endocytosis (Soulet et al. 2005), is involved in transporting MDVs and regulating immune responses involving activation of STING, TBK1 and IRF3/7. High levels of MDV‐linked SNX9 are associated with downregulation of IFNβ and IL‐6 (Soulet et al. 2005), two cytokines involved in immune response modulation and expression of interferon stimulated genes (Fan and Zhang 2013). Additionally, in OPA1 knockout cells, which exhibit impaired mitochondrial fragmentation and cristae structure, there is increased IL‐6 and packaging of the interferon stimulated gene IFIT1 into EVs (Todkar et al. 2021). This indicates that MDVs are involved in the initiation of innate immune responses. Conversely, ISGylation reduces EX release by decreasing the number of MVBs. Mechanistically, ISG15 conjugation triggered MVB co‐localization with lysosomes and promoted the aggregation and lysosomal degradation of MVB proteins (Villarroya‐Beltri et al. 2016). In addition, Parkin‐mediated mitophagy limits excessive ROS production and cytosolic mtDNA release from cells, thereby leading to inflammasome activation and IL‐1β and IL‐18 mediated pyroptosis (Freeman et al. 2017).

These findings suggest that MDVs trigger a defensive mechanism against pathogens. RIG‐I is an antiviral protein known to regulate mitochondrial immune responses upon, and is also carried by immune cell‐derived EVs to recruit additional immune cells (Daßler‐Plenker et al. 2016; Bek et al. 2019). However, a direct link between RIG‐I and MDV release has not been demonstrated. Similarly, MAVS, a mitochondrial antiviral signalling protein commonly associated with RIG‐I, is ubiquitinated by RNF34, triggering mitophagy and dampening innate immune responses during viral infection (He et al. 2019; Sun et al. 2016). This links the RIG‐I pathway to mitochondrial quality control. Certain bacteria can stimulate the generation of ROS‐containing MDVs, which are subsequently delivered to bacteria‐containing phagosomes. The process is IRE1a‐ and Parkin‐dependent, promoting the accumulation of H_2_O_2_ within MDVs to enhance antimicrobial activity. The accumulation of ROS also requires TLR2/4/9‐signalling and the presence of SOD2, a mitochondrial superoxide dismutase (SOD2) detected in MDVs (Abuaita et al. 2018). This mechanism is believed to represent a host defence strategy that enables macrophages to deliver mitochondrial effector molecules directly into phagosomes, thereby enhancing their antimicrobial function.

In macrophages, MDVs were able to prevent packaging of inflammatory proteins into EVs. Cells selectively separate proinflammatory cargo (e.g., damaged mtDNA and ubiquitinated proteins) from non‐inflammatory components during EV biogenesis (Todkar et al. 2021). The mitochondrial components found in normal EVs are typically non‐inflammatory, whereas proinflammatory cargo is usually prevented from packaging into EVs (Zhang et al. 2010). In this context, SNX9 promotes packaging of mitochondrial proteins into sEVs (Soulet et al. 2005), while Parkin redirects DAMPs to lysosomes, thereby limiting inflammation (Matheoud et al. 2016).

Another study has shown that EVs containing mtDNA and mitochondrial proteins can contribute to the induction of a proinflammatory response (Todkar et al. 2021). However, cells may actively avoid packaging such cargo into EVs due to the risk of extracellular release of ILVs via MVB fusion, which could exacerbate inflammation. Instead, this cargo is more likely directed into MDVs, which offers a shorter and more controlled route for degradation compared to the ILV‐MVB route (Todkar et al. 2021; Tamai et al. 2010). This selective routing is likely due to the proinflammatory nature of mitochondrial content, which can act as DAMPs. The packaging of mitochondrial content into EVs requires OPA1 and SNX9, while Parkin redirects mitochondrial DAMPs towards lysosomal degradation, thereby preventing inflammation (Todkar et al. 2021). Surprisingly, MDVs have been found to suppress inflammation by regulating the packaging of damaged mitochondrial proteins into EVs (Todkar et al. 2021). Under inflammatory conditions, packaging of proinflammatory mitochondrial cargo into small EX‐like vesicles favours inflammation (Todkar et al. 2021). These sEVs were Alix‐negative and CD9‐positive, and predominantly <150 nm—this suggests the possibility of both ESCRT‐dependent and ‐independent mechanism of EX release. However, SNX9 facilitates mitochondrial protein packaging into sEVs and is associated with SNX9‐dependent MDVs that can also regulate MitAp (Matheoud et al. 2016). This mechanism is blocked by Parkin, which redirects damaged mitochondrial cargo to lysosomes. The release of such cargo, including mtDNA and damaged mitochondria, can act as DAMPs and activate the innate immune system (Zhu et al. 2018; Ghiringhelli et al. 2009). Interestingly, EVs and MDV can also play a role in preventing mitophagy, offering alternative pathways for mitochondrial quality control (Phinney et al. 2015). Altogether, these findings reveal a MDV‐involved mechanism in which mitochondrial cargo is selectively packaged into EX‐like sEVs to modulate inflammation and maintain cellular homeostasis.

MDVs were shown to have a potential protective effect during hypoxic stress. Under hypoxic conditions, an increase of MDV release is negatively correlated with cardiomyocyte apoptosis. The administration of exogenous MDVs inhibited apoptosis in cardiomyocytes via Bcl‐2 interaction (Li et al. 2020). In a similar fashion, sEVs derived from mesenchymal stem cells, specifically Exs, can protect cardiomyocytes under pre‐ or post‐hypoxic conditions through reducing apoptosis (Della Rocca et al. 2023).

A vesicle release mechanism strikingly similar to MDV formation has been implicated in systemic lupus erythromatosus (SLE). In this autoimmune condition, neutrophils are unable to complete mitophagy under mitochondrial stress. Instead, neutrophils generate MDV‐like vesicles to remove damaged or oxidized mtDNA. However, in SLE, this MDV‐mediated transportation of oxidized mtDNA transportation to endolysosomes is disrupted, contributing to immune dysregulation and inflammation (Caielli et al. 2016). Neutrophils also release EVs in response to various stimuli, such as bacterial and pharmaceutical agents, independently of the release of neutrophil extracellular traps. These neutrophil‐derived sEVs modulate macrophage inflammatory responses and cytokine production (Allen et al. 2022), further highlighting the immunomodulatory potential of vesicle‐mediated communication. These findings suggest that MDVs may play a protective role in autoimmune disorders by facilitating the removal of proinflammatory mitochondrial components and preventing their release into the extracellular space.

Altogether, the evidence underscores a critical link between mitophagy, MDVs and the innate immune response. It would be of interest to further define the roles of MDVs in innate immune cells activation under mitophagy‐deficient conditions and to determine whether MDV cargo shuttling contributes to immune modulation when loaded with potential proinflammatory signalling molecules.

### 7.3 MDVs and Mitochondrial Antigen Presentation (MitAP)

MitAp is a critical process for initiating immune responses against abnormal or damaged cells. During MitAP, MDVs transport mitochondrial antigens to be processed and presented to immune cells, primarily via MHC Class I molecules. This process is conceptually similar to the role of sEVs in antigen presentation. The bacterial origin of the mitochondrion may explain how mitophagy‐mediated MitAP occurs on the cell surface (McLelland and Fon 2018). MitAP can be stimulated through the loss of PINK1 and exposure to the bacterial toxin lipopolysaccharide (LPS) or heat shock (Matheoud et al. 2016; McLelland et al. 2014). In contrast, treatment with the toxin carbonyl cyanide m‐chlorophenyl hydrazine (CCCP; a mitochondrial uncoupler that induces mitophagy) does not stimulate MitAP. Moreover, CCCP treatment following heat shock strongly inhibits MitAP. These findings suggest that mitophagy may suppress MitAP to prevent excessive immune activation and cell death (Matheoud et al. 2016; Fahmy et al. 2019). Mechanistically, PINK1 and Parkin suppress MDV formation and inhibit lysosome‐dependent MitAP. The loss of both PINK1 and Parkin led to the formation of MDVs that transport specific mitochondrial cargo to late endosomes, a process regulated by SNX9, RAB9 and RAB7 (Matheoud et al. 2016). Furthermore, Parkin deficiency results in impaired immune modulation, reduced endosomal tubulation and decreased levels of VPS35, an important vesicle sorting protein (Song et al. 2016). In vitro and in vivo studies demonstrate that the absence of PINK1 and Parkin led to elevated presentation of mitochondrial antigens on MHC Class I molecules in macrophages and DCs by a vacuolar pathway distinct from mitophagy (Matheoud et al. 2016; Sugiura et al. 2017). This supports the idea that MDV formation, rather than mitophagy, drives MitAP.

MDVs can regulate MitAP through delivering cargo from the mitochondria to other organelles (Matheoud et al. 2016). A central event in MitAP initiation is PINK1 phosphorylation, which leads to the dissociation of the endosomal adaptor TOLLIP from IRAK1 (Lee and Chung 2012). TOLLIP is a key regulator of the innate immune response via suppression of the IL‐1 pathway. TOLLIP is also essential for MDV uptake by lysosomes, particularly when TOM20 is present on the MDV surface (Ryan et al. 2020; Burns et al. 2000). TOLLIP also stabilizes STING, an ER‐associated protein involved in innate immunity, and prevents STING degradation through direct interaction. The TOLLIP‐STING interaction prevents lysosomal degradation in order to maintain immune homeostasis, which might be crucial in preventing neurodegenerative diseases. Indeed, TOLLIP has been shown to mediate the clearance of polyQ protein aggregates linked to Huntington's disease (Pokatayev et al. 2020).

MDVs are thus critical for presenting mitochondrial components via the MHC Class I at the cell surface. Whether MDVs also contribute to MHC Class II presentation remains unknown. It is hypothesized that once mitochondrial antigens are processed inside the lysosome, the mitochondrial peptides may be loaded onto MHC I molecules inside the ER. In Parkinson's disease, the accumulation of TOLLIP along MDV trafficking routes impairs the stabilization of STING. Mitochondrial stress is alleviated when Parkin is sequestered, thereby restoring regulation of STING and MitAp (Ryan et al. 2020; Ryan and Tumbarello 2021). However, the precise mechanisms linking MDV trafficking, lysosomal processing and ER‐mediated antigen presentation remain to be fully elucidated. Furthermore, it would be of interest to further investigate the role of MDVs in TOLLIP‐STING interaction, as MDVs are a less lytic route alternative to mitophagy.

## 8 Translational Applications and Challenges in MDV Isolation and Separation

The potential of EVs, particularly sEVs, in early disease detection and therapeutic applications is well established (Welsh et al. 2024; Kalluri and LeBleu 2020). sEVs are increasingly integrated into translational medicine as diagnostic biomarkers and therapeutic delivery vehicles. In contrast, MDVs remain largely confined to basic research despite their emerging relevance in diseases and disorders. MDVs represent a minor fraction of the total EV population in biofluids and the size range overlaps with other sEVs, making MDVs difficult to distinguish among other EVs. However, MDVs carry unique mitochondrial biomarkers, such as Parkin, Pink, mtDNA, SNX9 and TOM proteins, that could be leveraged for early detection of malignant, autoimmune, neurodegenerative, cardiac and liver disorders (Matheoud et al. 2016; König and McBride 2024; Soubannier, Rippstein, et al. 2012; Ryan et al. 2020; Tang et al. 2025; Gagliardi et al. 2024). Under oxidative stress, MDVs selectively encapsulate damaged mitochondrial cargo, which may signal pathology in neurodegeneration or serve as an early indicator in cancer (Towers et al. 2021; Gagliardi et al. 2024). From a diagnostic perspective, the MDV/mitophagy balance could serve as a biomarker of mitochondrial inflammation, with implications for diseases involving metabolic dysfunction, immune dysregulation and cellular stress. Despite the promise, MDVs have yet to be translated into clinical applications, partly due to challenges in isolation, engineering and characterization. However, there is the potential for selective cargo loading and the use of MDVs as mitochondrial‐targeting vesicles for mtDNA repair, delivery of pharmacological, modulating immune responses and inducing intrinsic mitochondrial‐mediated apoptosis (Horbay and Bilyy 2016; König and McBride 2024; Todkar et al. 2021; Abuaita et al. 2018; Ryan and Tumbarello 2021; Kroemer and Martin 2005).

The overlapping size of MDVs and sEVs complicates their separation using standard protocols such as ultracentrifugation and ultrafiltration, which co‐isolate both vesicle types. Moreover, MDVs are often targeted to lysosomes for degradation due to their ubiquitylated and SUMOylated cargo, reducing their availability for extracellular release (Roberts et al. 2016; Geisler et al. 2010). The impact of isolation methods on the surface corona of MDVs, a potential analogue to the EV corona, remains poorly understood but may influence vesicle function (Buzas 2022).

A proposed strategy for distinguishing MDVs from sEVs involves biomarker profiling. While CD9, CD63 and CD81 are classical sEV markers, mitochondrial respiratory complexes I–V, Parkin and PINK1 are indicative of MDVs (Picca et al. 2019). Following MISEV 2018 guidelines (Théry et al. 2018), the authors suggested combining western blotting, mtDNA quantification, and immunolabelling of MDV‐specific proteins (e.g., TOM20, PDH, VDAC1, Core2) to confirm MDV presence (Picca et al. 2019). However, complete separation remains elusive. sEV isolation methods, such as size‐exclusion chromatography, immunoaffinity capture, bead‐based separation and ceramide‐blocking (Théry et al. 2018; Yu et al. 2018), may still co‐isolate MDVs. Moreover, MDV enrichment often requires mitochondrial disruption, risking vesicle damage and contamination. Currently, there is no proven method to separate these two fractions of vesicles, but the rapidly growing interest in MDV research could change this 1 day as several methods have shown selectivity in inhibiting or boosting the biogenesis and release of MDVs or sEVs.

Several studies have explored modulating vesicle populations by targeting key biogenesis pathways. For instance, inhibiting MDV markers (e.g., TOM20, PDH, DRP1) while treating cells with EV‐releasing agents (e.g., bortezomib, carfilzomib, melphalan) increases sEV output while suppressing MDV formation (Soubannier, McLelland, et al. 2012; Rosdah et al. 2022; Bandari et al. 2018). On the other hand, xanthine oxidase/xanthine treatment or a subtoxic dose of ROS‐generating enzyme glucose oxidase can increase PDH⁺ and TOM20⁺ MDVs by 2–3 fold (Soubannier, McLelland, et al. 2012). In addition, EV‐targeting agents such as PDZ1i (Syntenin‐1 inhibitor), manumycin (ESCRT‐dependent inhibitor) and GW4869 (ESCRT‐independent inhibitor) block sEV release but may not affect MDV production (Essandoh et al. 2015; Pradhan et al. 2021; Datta et al. 2017). The targeting of specific components of the ESCRT‐dependent and ‐independent pathways would also block sEV release and thereby boost MDV numbers (Wei et al. 2021; Datta et al. 2017). However, this has yet to be studied. Lastly, boosting MDV numbers can be accomplished using the protonophore CCCP to globally depolarize mitochondria and induce Parkin‐dependent mitophagy (Sugiura et al. 2014).

The detection and characterization of MDVs can be accomplished via several ways. Immunoblotting and fluorescence microscopy using Mitotracker Red 633 or fluorescent tagging of Syntenin‐1, RAB31 or tetraspanins can help identify vesicle origin (Lötvall et al. 2014; Soubannier, McLelland, et al. 2012; Wei et al. 2021; Kashyap et al. 2021). Further approaches to separately study sEVs and MDVs can be via the use of lysosomal inhibitors (e.g., Bafilomycin A) or electron transport chain disruptors (e.g., Antimycin A) as these agents can modulate MDV release and provide insights into distinct vesicle pathways (Soubannier, McLelland, et al. 2012).

## 9 Conclusion

As descendants of bacteria, mitochondria retain the ability to generate their own intracellular vesicles, known as MDVs. MDVs and sEVs share several characteristics: both utilize the endosomal packaging system and rely on lysosomal degradation pathways. However, MDVs have the unique ability to be trafficked not only to lysosomes but also to peroxisomes, either via MVBs or through direct transport. MDVs offer an alternative to the more lytic process of mitophagy by providing a safer mechanism for the selective removal of damaged mitochondrial components. Both MDVs and sEVs encapsulate damaged or targeted cargo, package the cargo within vesicles, and direct the cargo either towards lysosomal degradation or extracellular release. Disruption of these vesicular pathways has been implicated in various diseases, including neurodegenerative disorders and cancers. Moreover, MDVs and sEVs play important roles in immunity and inflammation. They can carry immunostimulatory molecules and pathogen‐derived antigens, contributing to immune activation. Finally, MDVs and sEVs serve as carriers of disease biomarkers such as Parkin and PINK1, making them valuable tools for diagnostics and potential targets for therapeutic intervention. Despite their similarities, distinguishing between MDVs and sEVs remains challenging. Nevertheless, significant progress is being made in advancing our understanding and separation of these vesicle populations.

## Author Contributions

**Rostyslav Horbay**: conceptualization, methodology, writing – original draft, formal analysis, writing – review and editing, validation, data curation, visualization, investigation, supervision, project administration, software, resources. **Vasyl Syrvatka**: writing – original draft, conceptualization, data curation, formal analysis, visualization, methodology, investigation, writing – review and editing, software, validation. **Artem Bedzay**: conceptualization, data curation, formal analysis, visualization, writing – original draft, methodology, investigation, software, validation, writing – review and editing. **Mikaela van der Merwe**: conceptualization, data curation, formal analysis, visualization, writing – original draft, methodology, investigation, writing – review and editing, software, validation. **Dylan Burger**: conceptualization, data curation, formal analysis, visualization, writing – original draft, methodology, investigation, writing – review and editing, software, validation. **Shawn T. Beug**: resources, funding acquisition, validation, software, methodology, investigation, supervision, project administration, writing – review & editing, writing – original draft, visualization, formal analysis, conceptualization, data curation.

## Funding

This work was supported by Cancer Research Society (#935202) and Canadian Institutes for Health Research (#PJT‐169126).

## Ethics Statement

The authors have nothing to report.

## Conflicts of Interest

The authors declare no conflicts of interest.

## Data Availability

Data sharing not applicable to this article as no datasets were generated or analysed during the current study.
