# Correction to “From Mitochondria to Immunity: The Emerging Roles of Mitochondria‐Derived Vesicles and Small Extracellular Vesicles in Cellular Communication and Disease”

**DOI:** 10.1002/jev2.70212

**Published:** 2025-12-08

1

Horbay, R., V. Syrvatka, A. Bedzay, M. van der Merwe, D. Burger, and S. T. Beug. 2025. “ From Mitochondria to Immunity: The Emerging Roles of Mitochondria‐Derived Vesicles and Small Extracellular Vesicles in Cellular Communication and Disease.” *Journal of Extracellular Vesicles* 14, no. 11: e70192. https://doi.org/10.1002/jev2.70192

In the originally published article, in the figure captions, numbers that denoted parts of the figures were mistakenly given as references. The figures with their correct captions are shown below.

We apologize for this error.


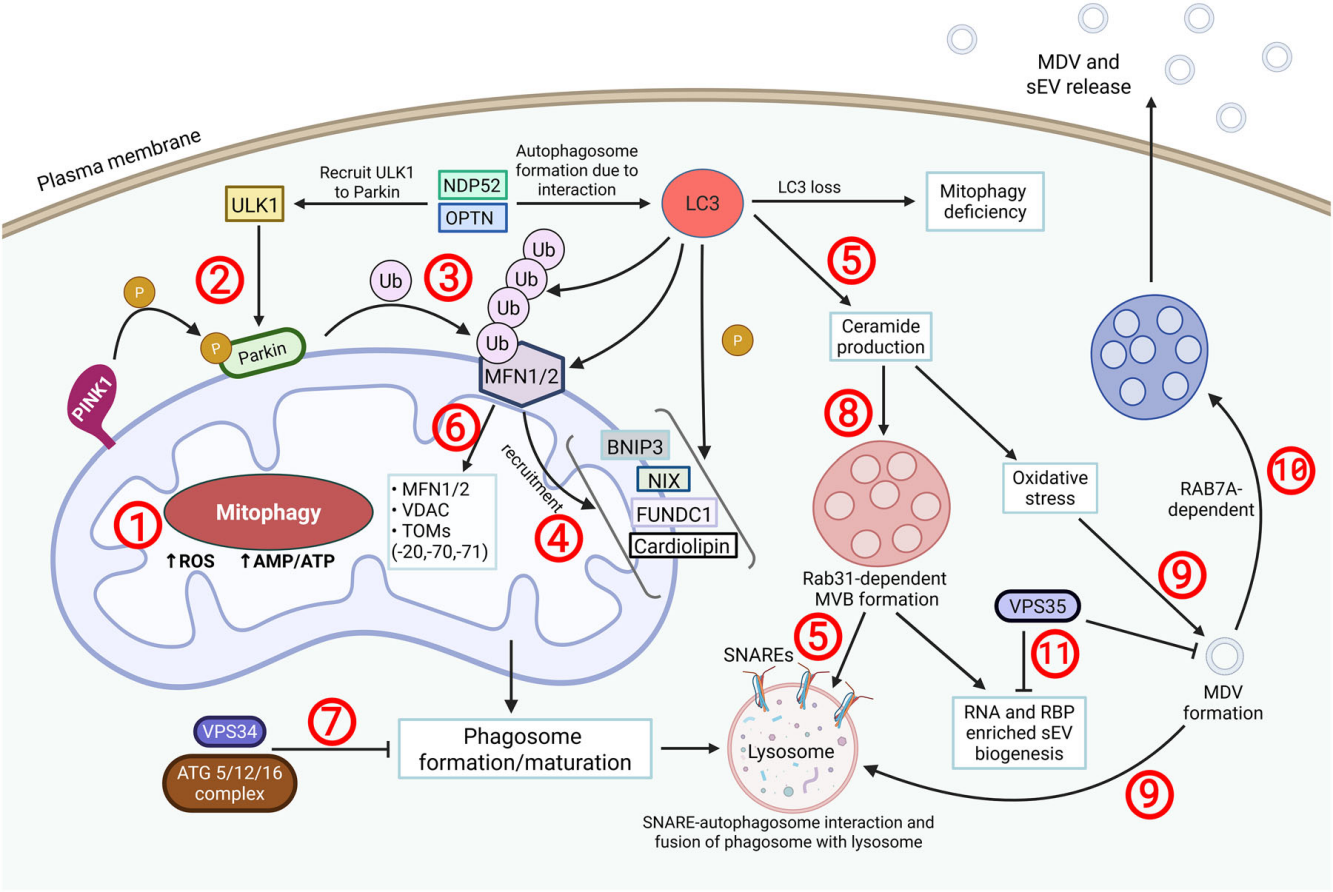


**FIGURE 1** Mitophagy and MDVs maintain mitochondrial dynamics. To prevent excessive ROS accumulation, mitochondria initiate mitophagy following the loss of electrochemical mitochondrial membrane potential (1). Mitophagy begins with PINK1‐mediated phosphorylation of Parkin (2) and subsequent MFN1/2 ubiquitination (3). MFNs recruit BNIP3, NIX, FUNDC1 and Cardiolipin to target damaged mitochondria, while LC3 will phosphorylate these four receptors (4). LC3 is a key player in mitochondrial quality control by promoting the engulfment of defective or damaged mitochondria into phagosomes, including via the MDV pathway (5). LC3 activity is regulated by NDP52 and OPTN, which recruit ULK1 to Parkin, enabling further Parkin‐mediated ubiquitination and phosphorylation of MFN1/2 (2,3), VDAC and TOM20 (6). If not inhibited by the VPS34 and ATG5/12/16 complex, the phagosome is trafficked to the lysosome for degradation (7). Loss of LC3 results in mitophagy deficiency, impaired ceramide retention, which requires RAB31, will disrupt MVB formation (8), oxidative stress, and MDV formation. Most MDVs are degraded in lysosomes or peroxisomes (9). However, a subset can be packaged into MVBs and released extracellularly via RAB7‐dependent trafficking of the MDV towards the MVB (10). On the other hand, VPS35 can inhibit both MDV and sEV biogenesis (11).


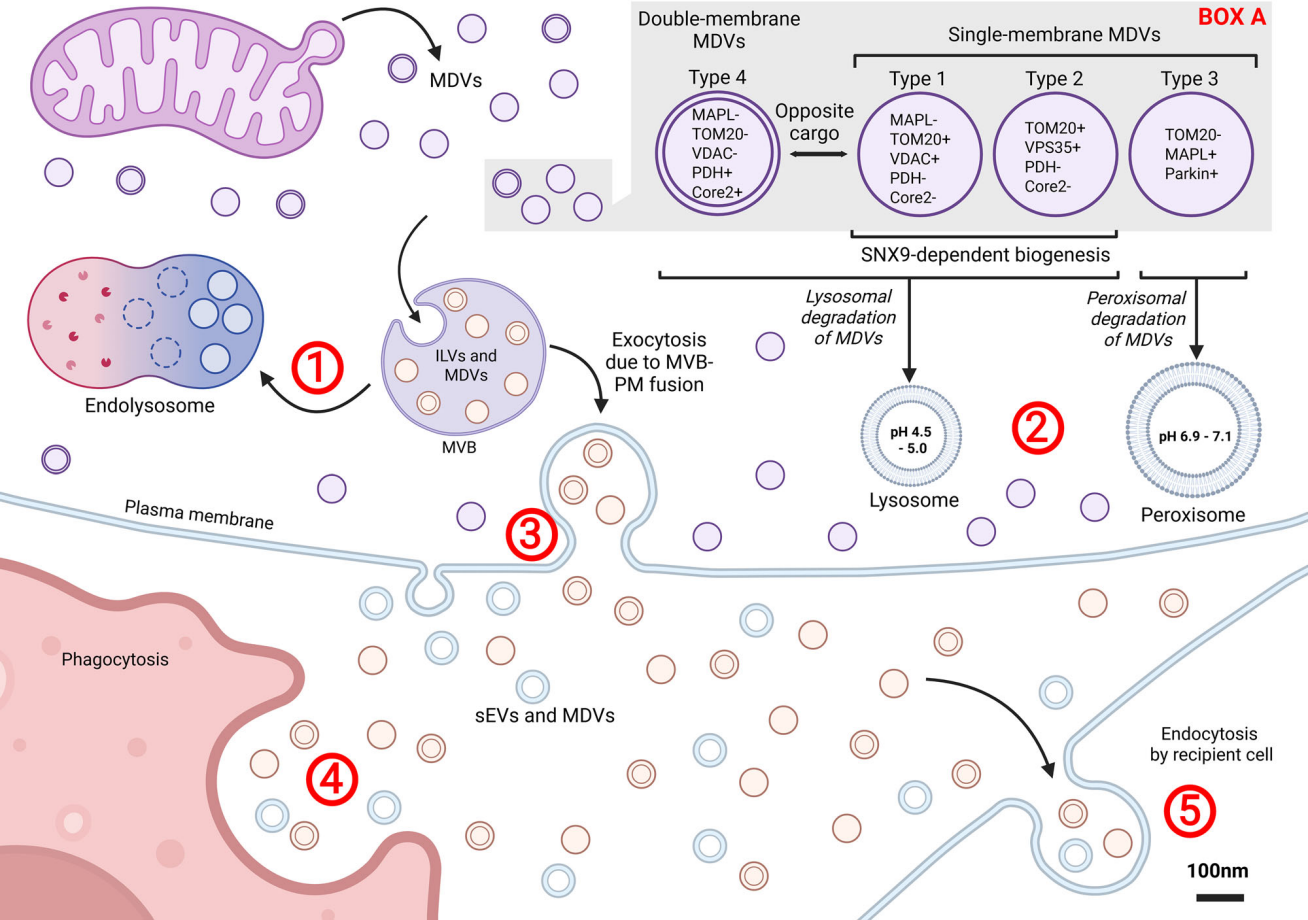


**FIGURE 2** Types of MDVs. There are four main types of MDVs that are classified in accordance with cargo content and membrane structure. Types 1–3 are single‐membrane, while Type 4 is double‐membrane (Box A). Depending on the cargo content, MDVs are directed to lysosomes or peroxisomes for cargo degradation. SNX9 is involved in the biogenesis of Type 1 and Type 2 MDVs. The classification is based on the presence or absence of specific proteins: Core2, MAPL, PDH, TOM20, VDAC and VPS35. MDVs may enter endosomes, which later fuse with lysosomes for degradation (1). Alternatively, MDVs can be incorporated into MVBs and degraded by lysosomes or peroxisomes (2) or escape the cell in an EV‐like manner via plasma membrane‐MVB fusion (3). In this case, both MDVs and sEVs can be endocytosed/phagocytosed by recipient cells such as antigen‐presenting cells (4,5).


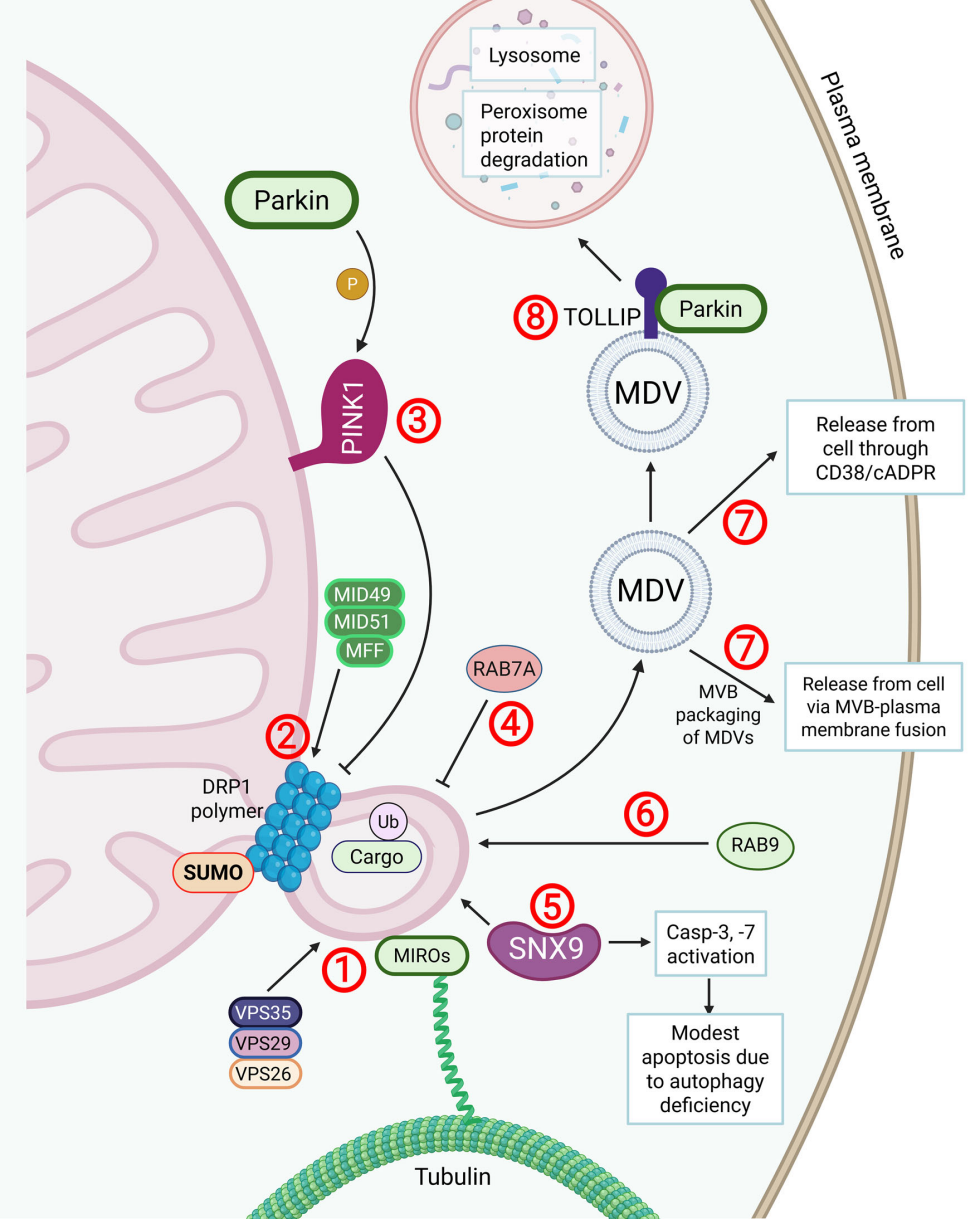


**FIGURE 3** The role of SNX9 and DRP‐1 in MDV release. MDV formation begins with membrane curvature initiation where VPS35, a core component of the VPS35/VPS29/VPS26 retromer complex, plays a key role, and MIRO proteins guide tubulation (1). MID49, MID51 and MFF proteins direct DRP1, a mitochondrial fission protein involved in mitochondrial biogenesis, to polymerize and form a neck around the budding MDV (2). RAB7A and PINK1/Parkin can inhibit DRP1‐dependent MDV formation (3,4). SNX9 acts as an adaptor protein essential for MDV formation, lysosomal delivery of MDVs, mitochondrial cargo packaging into sEVs, and caspase activation (5). In contrast, RAB9 promotes DRP1‐mediated MDV release (6). Once formed, MDVs can be released from the cell via MVBs or through a CD38/cADPR dependent process (7), though most are directed to lysosomes or peroxisomes for cargo degradation. Tollip coordinates the trafficking of damaged cargo into MDVs for degradation (8).


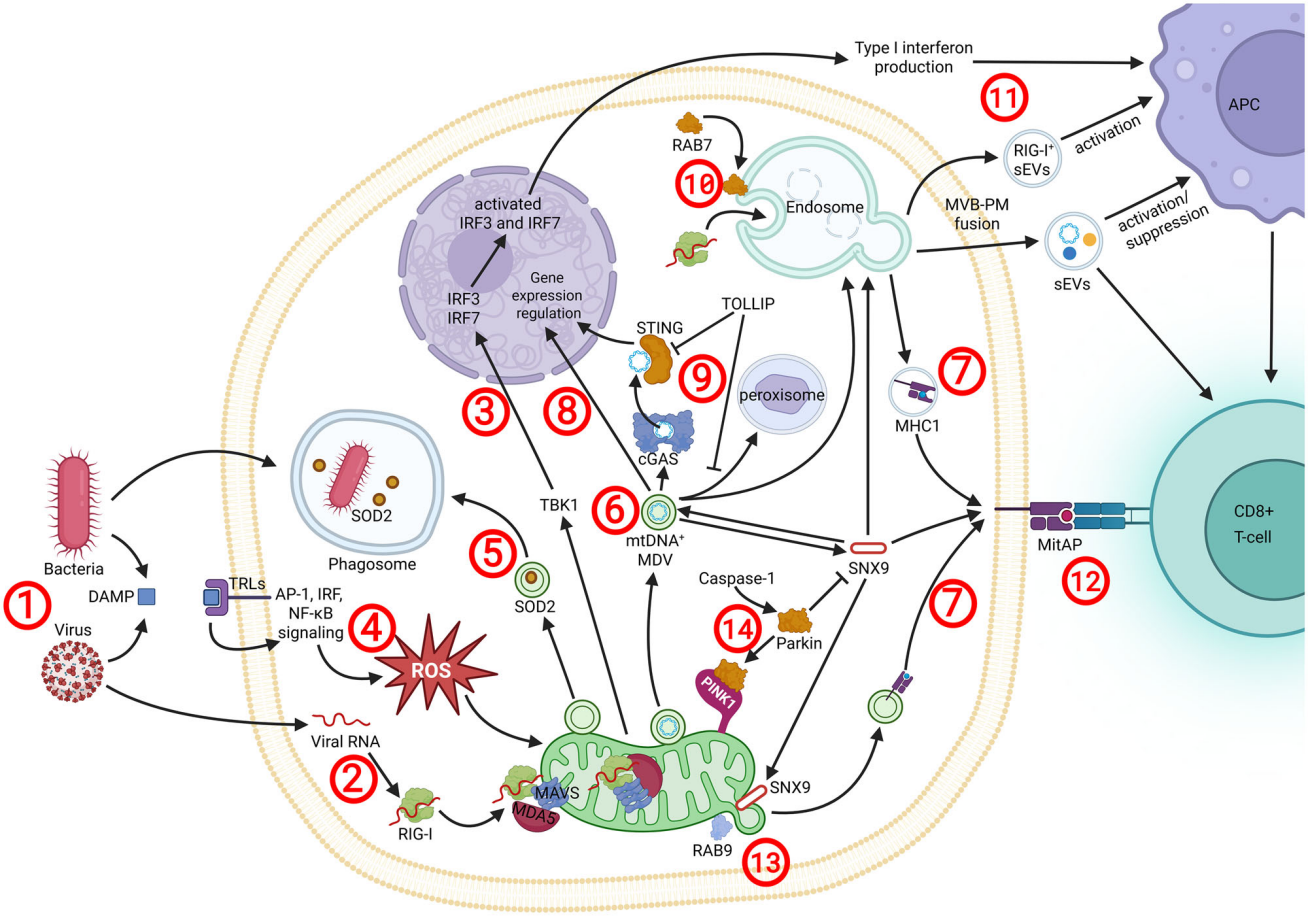


**FIGURE 4** The role of sEVs and MDVs in immunity. Bacteria and viruses can trigger immune responses via MDVs and sEVs (1). Viral RNA activates RIG‐I, initiating the RIG‐I‐MDA5‐MAVS complex (2), which activates TBK1 and leads to Type I IFN production via IRF3/7 activation (3). Bacterial and viral DAMPs activate TLRs, leading to ROS accumulation (4) and MDV release. These MDVs could carry bacteria‐targeting SOD2 (5), mtDNA (6), and MHC‐I molecules (7). mtDNA‐containing MDVs can regulate gene expression (8), initiate immune responses through the cGAS‐STING pathway (9), or be degraded via peroxisomes (9). The last two events can be inhibited by TOLLIP (9). Sensor proteins, such as RIG‐I, are packaged into sEVs in a RAB7‐dependent manner (10), and these sEVs can activate antigen‐presenting cells and modulate T cell responses (11). T cells can also be activated via MitAP (12). Proteins involved in EV biogenesis, such as RAB9 and SNX9, facilitate MDV release and antigen presentation to T cells (13). Notably, SNX9, which is essential for MDV release and MitAP, can be inhibited by Parkin and PINK1. To reduce mitophagy, Parkin can be inactivated via Caspase‐1 cleavage (14).

